# Systematic review of the management of incontinence and promotion of continence in older people in care homes: descriptive studies with urinary incontinence as primary focus

**DOI:** 10.1111/j.1365-2648.2010.05481.x

**Published:** 2011-02

**Authors:** Brenda Roe, Lisa Flanagan, Barbara Jack, James Barrett, Alan Chung, Christine Shaw, Kate Williams

**Affiliations:** Evidence-based Practice Research Centre, Faculty of Health, Edge Hill UniversityUK, and Honorary Fellow Personal Social Services Research Unit, University of ManchesterUK; Royal Liverpool and Broadgreen University Hospital NHS TrustLiverpool, UK; Evidence-based Practice Research Centre, Edge Hill UniversityUK, and Head of Research and Scholarship Faculty of Health, Edge Hill UniversityUK; Department of Geriatric and Stroke Medicine, Wirral University Teaching Hospitals NHS Foundation TrustWirral, UK, and Evidence-based Practice Research Centre, Faculty of Health, Edge Hill UniversityUK; Birmingham Own Health, NHS DirectBirmingham, UK; Department of Care Sciences, University of GlamorganUK; Department of Health Sciences, University of LeicesterUK

**Keywords:** care homes, incontinence, nursing, older people, systematic review

## Abstract

**Aim:**

This is a review of descriptive studies with incontinence as the primary focus in older people in care homes.

**Background:**

Incontinence is prevalent among residents of care home populations.

**Data sources:**

MEDLINE and CINAHL were searched from 1996 to 2007 using the highly sensitive search strings of the Cochrane Incontinence Review Group for urinary and faecal incontinence including all research designs. Search strings were modified to enhance selectiveness for care homes and older people and exclude studies involving surgical or pharmacological interventions. Searching of reference sections from identified studies was also used to supplement electronic searches. The Cochrane Library was searched for relevant systematic reviews to locate relevant studies from those included or excluded from reviews. The search was limited to English-language publications.

**Methods:**

A systematic review of studies on the management of incontinence, promotion of continence or maintenance of continence in care homes was conducted in 2007–2009. This is a report of descriptive studies.

**Results:**

Ten studies were identified that reported on prevalence and incidence of incontinence (urinary with or without faecal), policies, assessment, documentation, management or economic evaluation of its management. Use of incontinence pads and toileting programmes comprised the most common management approaches used. No studies were identified that attempted to maintain continence of residents in care homes.

**Conclusions:**

Studies on maintaining continence and identifying components of toileting programmes that are successful in managing or preventing incontinence and promoting continence in residents of care home populations along with their economic evaluation are warranted.

## Introduction

Incontinence is a prevalent condition affecting older people in community and institutional settings. Incontinence is estimated to affect from 31% to 70% of older people in care homes and incurs personal and institutional costs related to staff time, laundry, aids and appliances (McGrother *et al.* 2003, Fonda *et al.* 2005). A systematic review was undertaken to identify empirical research for the management of incontinence, promotion of continence or maintenance of continence in older people in care homes to describe and inform practice and future research. The overall review identified intervention studies and descriptive observational studies (as defined by Rawlins 2008) as the basis of an umbrella review. The review of interventions looking at effectiveness has been reported elsewhere due to its focus and number of studies available (under review). The purpose of this study is a report of descriptive studies that included urinary incontinence (UI) or continence status and its management or an economic evaluation as the primary focus.

## Background

Clinical guidelines for the management of incontinence (Fantl *et al.* 1996, Button *et al.* 1998, NICE 2006, 2007), international consultation conferences (Abrams *et al.* 2009), systematic reviews (Eustice *et al.* 2000, Ostaszkiewicz *et al.* 2004a,b;, Wallace *et al.* 2004) and metastudy (Roe *et al.* 2007a,b;) have reviewed the evidence to inform and guide the management of incontinence. Incontinence has been defined as ‘the involuntary or inappropriate passing of urine and/or faeces that has an impact on social functioning or hygiene. It also includes nocturnal enuresis (bed wetting)’ (DH 2000, p. 7). The term care homes is generic and describes institutional settings that provide long term care for older people and includes nursing homes (giving nursing care), residential care homes (giving mainly social care) or mixed (giving both).

Management techniques are largely aimed at hospital or community populations. Research on the management of incontinence in care home populations has been undertaken, predominantly in the United States of America (USA) by designated research teams (for example, Schnelle *et al.* 1989, Colling *et al.* 1992, Ouslander *et al.* 1995) and their findings may not be transferable to other populations or settings where the organization, staffing and delivery of care may vary. Research on long-term follow-up to indicate if practices are sustained is generally unavailable. The focus of research has been on the management of incontinence whereas the maintenance of continence for older people that enter care homes has not featured.

## The review

### Aim

This study presents the review of descriptive empirical studies on the management of incontinence, promotion of continence or maintenance of continence in people aged 65 years and above in care homes with UI as the primary focus.

### Objectives

The objective of this study is to identify care practices for the management of incontinence, promotion of continence or maintenance of continence in older people in care homes, and to provide a narrative synthesis of study designs, methods, findings and outcomes.

### Design

A systematic review of studies that used quantitative or qualitative designs and methods was undertaken to provide a narrative synthesis. The PRISMA statement (formerly QOUROM) was used as a guide (Moher *et al.* 2009).

### Search methods

Electronic searches were the prime method employed using MEDLINE and CINAHL via OVID (January 1966 to February 2007) to locate published studies (in English). Relevant studies awaiting assessment from updated searches to May 2010 are available (see supporting information Table S1 in the online version of the article in Wiley Online Library). Hand searching of reference sections from yielded studies and targeted journals for relevant references identified supplemented the electronic searches. The Cochrane Library was also searched for relevant systematic reviews to locate relevant studies from those included or excluded from reviews.

#### Search strategy

The MEDLINE highly sensitive search strings from the Cochrane Incontinence Review Group for UI and faecal incontinence (FI) were adopted and included all empirical designs (Grant *et al.* 2006). Search strings were modified to enhance the selectiveness for care homes and older people and exclude studies involving surgical or pharmacological interventions. Copies of the search strategies are available from the lead author.

#### Inclusion criteria

Empirical studies of the management of incontinence, promotion of continence or maintenance of continence in older people aged 65 years and above in care homes were located. Studies of medical or behavioural approaches with incontinence defined or specified were included (see Table 1).

**Table 1:** Inclusion criteria for empirical studies

Studies were accepted that met inclusion criteria according to study type, participants' age, setting, types of conditions, types of interventions, language and date of the publication and the availability of articles. Included studies met all the following criteria
1. Studies with ‘older people’ residents/participants aged 65 or over or a majority with a mean age of 65 and over living in care homes, residential homes, nursing homes or assisted living facilities
2. Empirical studies that included descriptive, observational or interventions (which include nursing, medical or behavioural interventions) aimed at the management of incontinence or the promotion or maintenance of continence. Study designs included randomized-controlled trials, quasi-randomized trials, case–control studies, before and after studies, cohort studies, survey, economic evaluation or empirical studies
3. Outcome measures that include continence status and management of incontinence, promotion of continence or maintenance of continence
4. *Type of condition*. Specified urinary incontinence or urinary and faecal incontinence with or without a definition included
5. *Language*. Published articles in English. Studies published in languages other than English will only be accepted if English translation is available
6. Year of publication

#### Exclusion criteria

Studies of pharmaceutical or surgical interventions were excluded as the focus was on care practices undertaken by nurses or care assistants in care homes. Non-empirical studies were excluded (see Table 2).

**Table 2:** Exclusion criteria

Studies or articles with any of the following elements were excluded from the review
1. Study type: Publications based on opinions of experts or level 5 non-empirical evidence
2. Participants: No mention of participants' ages, either as actual ages or means. Studies were also excluded if the mean age of participants was below the age of 65
3. Type of conditions: Studies where the primary outcome measures were not related to continence maintenance, continence promotion or the management of incontinence
4. Types of interventions: Studies that involved surgical or pharmacological interventions
5. Setting: If the study was conducted in hospital, participants' home, rehabilitation facilities, ‘care in the community’ or ‘step-down’ beds. Studies were excluded if participants only attend the nursing homes, residential homes, care homes or assisted living facilities on a day case basis or were not residents
6. Language: Studies were not published in English or no English translations could be obtained
7. Availability of articles: Studies were excluded if all available means were exhausted in locating the full article, that included electronic search, hand search, direct communication with the author or requisition from the British Library

### Search outcome

**Figure 1:**
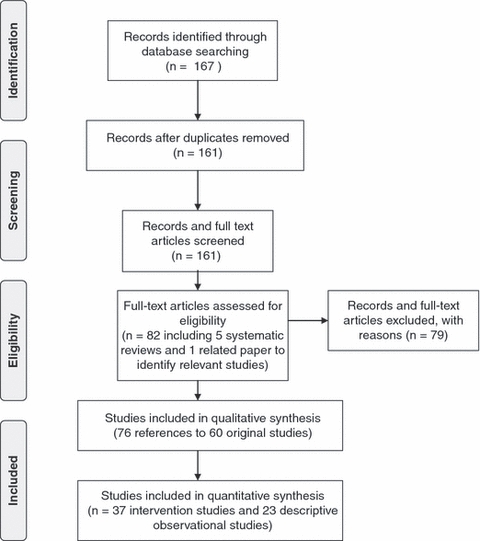
PRISMA 2009 flow diagram of the screening process.

There were 167 located references; 6 duplicates, 79 excluded studies (see supporting information Table S2 in the online version of the article in Wiley Online Library) and 82 relevant references – five systematic reviews (all four Cochrane Reviews, Eustice *et al.* 2000, Ostaszkiewicz *et al.* 2004a,b;, Wallace *et al.* 2004; and one associated paper, Ostaszkiewicz *et al.* 2005) and 76 included references to 60 original studies (37 intervention studies and 23 observational/descriptive studies) (Figure 1).

### Quality appraisal

All, titles, abstracts and references identified were checked by two reviewers. Of the 60 original studies, all incorporated quantitative methods with the exception of one that used grounded theory and qualitative data (Robinson 2000). Quality assessments of all studies were undertaken by two reviewers using an 18-item checklist for quantitative studies and a 15-item checklist for qualitative studies. These checklists, used by Shaw *et al.* (2009, pp. 9–11) and adapted from Downs and Black (1998) and Kmet *et al.* (2004) were made relevant to this review. The checklist allowed a common approach to the assessment of study quality, strength of evidence and a total score derived. No studies were excluded based on quality appraisal.

### Data extraction

A data extraction form was developed, circulated for comment and agreement among co-reviewers. Electronic versions were used independently by three reviewers to extract information. This information was checked by a second reviewer and agreement reached for accuracy for all included studies. Data were extracted for study settings, purpose, populations and samples, methods, main findings and conclusions (Table 3) and synthesized in 2007–2009.

**Table 3:** Descriptive observational studies of urinary incontinence as the primary focus

Study	Setting	Purpose	Population and sample	Methods	Main outcomes/findings	Conclusions/notes	Quality rating
**Ouslander 1982**, descriptive	USA; 7 NH	Descriptive survey of population and care	954 total; 88% (842) included; majority >65 years of age. 419 (50%) with <UI – mean age 88·7 years, 73% (305) women; 27% (113) men	Quantitative. UI/FI. UI defined Documentary review medical records. Data on demography, major diagnoses, medication, diagnostic studies, complications, continence status on admission, mental status questionnaire (MSQ).	Most residents incontinent on admission (64%) 72% >1 incontinent episode/day or a catheter, with concomitant FI in 64%. 71% (299) frequent UI; 29% (120) occasional UI. Substantive cognitive impairment with MSQ score <3 and limitations of mobility related to severity of incontinence. 45% had complications with skin irritation or UTI. <15% of residents had incontinence recorded in NH records or efforts made to evaluate it.	Most residents had 1 often 2 diagnoses that may be related to incontinence-dementia (45%), stroke (28%), Parkinson disease (9%) and depression (6%).	15/28 54%
**Palmer 1991**, descriptive	USA; 8 NH operated by same corporation	Identify non-urologic risk factors for continence outcomes 1 year after admission	434 admissions; 196 (45%) remained at 1 year; mean age 82 years, sd 7. Majority women 77% (336); men 23% (98) Loss to follow-up; 96 (22%) died, 134 (31%) discharged, 8 (2%) with drew	Quantitative. UI defined-any reported daytime UI. Retrospective secondary data analysis. On mental morbidity on NH experience. Data collected by interview 2 weeks; 2 months and 1 year. Nursing assistants also interviewed on incontinence during day time UI presence or absence 7 am–7 pm. Data collected 1987–1988	UI associated with dementia (*r* = 0·26, *P*< 0·001), dependent walking (*r* = 0·22, *P*< 0·001), dependent transfer (*r* = 0·4, *P*< 0·001) at 2 weeks and significantly poorer adjustment to NH *t* = −4·38 (*P* < 0·001). 39% (167/434) UI at 2 weeks; 37% (138/317) UI at 2 months, 44% (84/196) UI at 1 year (χ^2^, *P*< 0·001) with 90% having dementia. Rate of new cases of UI over year was 27% (30/112) with men having a higher incident case of UI than women (9/16 vs. 21/96, χ2, *P*< 0·004). Dementia highly prevalent with new cases of UI (27/30, 90%). No change in continence status for 73% (130/178) of sample comparing 2 weeks and 1 year.	Variables significantly associated with UI at 1 year were UI at 2 weeks (*r* = 0·40), behavioural adjustment at 2 weeks (*r* = 0·26), gender (*r* = 0·24), mobility (*r* = 0·21) and a dementia diagnosis at 2 months(*r* = 0·18) (all (*P*< 0·05). UI increased significantly over 1 year (*P*< 0·001). Age was not associated with incidence or prevalence. Multiple regression analysis found risk factors for UI at 1 year were; male (68% highest factor); UI at 2 weeks (107%), poor behavioural adjustment to NH at 2 weeks (112%); dementia at 2 months (122%). A subject with these attributes was at a cumulative increased risk of having UI at 122%. Those with no detectable mental morbidity had the lowest incidence of UI.	19/20 95%
**Peet 1996**, descriptive	UK; random samples of 68 CHs (85% response rate)	Survey to assess nature of incontinence, management of UI, identify changes required, strategies used and if support was required by CHs	A random selection of 2 residents per CH; *n* = 96 (70% response rate); mean age 84·3 years; 81% (78) women, 19% (18) men	Quantitative. UI Methods of randomization unspecified. Retrospective documentary analysis. Assessment of individual residents. Unclear. Data collected on UI, severity, physical dependency, policies and practice. Data collected 1990.	69% (66) had UI most days with 39% (37) having severe symptoms; 85% (82) had mobility problems. Management of UI policies; 87% (62) use pads, continence sheets 62% (44), personalized bathing 51% (33), use of appliances or catheters 41% (29). 21% (15) had a policy that precluded admission of residents with UI. Promotion of continence policies; day time toileting 83% (59), aids 68% (48), night toileting 52% (37), use of toilet signs 49% (35), fluid restriction 38% (27). Current techniques used for 57% (55 residents); toileting 46% (44), fluid adjustment 16% (15), BT 6% (6), PFME 0. Need for continence service input/advice to change residents management of UI; pads 25% (24), drug therapy 15% (14), use of appliances 9% (9); change promotion of continence; investigations 47% (45), BT 31% (30), toileting 27% (26), use of aids 12% (11), fluids advice 9% (9). 57% (39) of homes requested more help with continence care.	No information provided on randomization or the reliability and validity of methods of data collection. Based on the findings, CHs and residents would benefit from further information and advice on how to manage UI. Nearly half the residents assessed warranted further investigations and nearly a third required a change to involve the use of BT or toileting programmes.	14/24 58%
**Sgadari 1997**, descriptive	Denmark, France, Iceland, Italy, Japan, Sweden, USA; 7 national samples from NH populations	Cross-national survey to report prevalence of UI, describe associated factors, compare the use of incontinence related tests and care practices.	279,191 elderly residents; Denmark (1799), France (167), Iceland (377), Italy (586), Japan (539), Sweden (436), USA (126,070). More than 40% of samples were aged 85 years and above, except Japan (35%). The majority were women 71% (198,941), men 29% (80,250)	Quantitative. UI defined 2 times/week How samples obtained not specified. State data for USA, Denmark and Iceland are population based. The remaining countries likely representative not as broadly based. Retrospective documentary analysis data from Minimum Data Set (MDS) and Resident Assessment Instrument (RAI). Cognitive Performance Scale (CPS >2 cognitive impairment); activities of daily living (ADLs); Case Mix Index (CMI), the higher a score the heavier a case mix and resource use; tests and care. practices. Statistical significance relates to chi-squares calculated separately for each country.	Prevalence of UI ranged from 42·9% (Japan) to 65·2% (France); FI from 22·4% (Denmark) to 55·5% (France); Mixed UI/FI from 20·5% (Denmark) to 52% (France). A significant positive association between increasing age and UI was found in all countries (Denmark *P*< 0·002; France *P*< 0·0007; Italy *P*< 0·003; Japan *P* < 0·002; USA *P* < 0·001) except Sweden (NS) and a negative association was found in Iceland (*P*< 0·0007). A strong association between dependent mobility and UI found across all countries (*P*< 0·0001) with those bedfast more likely (*P*< 0·0001; except in Sweden, NS). UI more prevalent in those with cognitive impairment (CPS >2) (*P* < 0·0001). Variation in testing those with UI for UTI with 48% having this in France but only 11% in Sweden and Italy. Faecal impaction was checked in 7·2% of UI residents in Iceland (*P* < 0·005) and 27·3% in France (*P*< 0·0001). Scheduled toileting was the lowest in Italy (11%) and the highest in Iceland (50·7%) (Japan 24·5%, Sweden 27·1%, France 28·7%, USA 35·1%) (*P*< 0·0001). Of note, Iceland commonly used scheduled toileting in non-incontinent residents (35·5%). Use of pads and briefs were highly favoured, ranging from 71·6% in USA to 90·1% in Denmark (*P*< 0·0001). A minority of residents with UI did not use toilets in France (4·4%) whereas a majority did not use them in Japan (52·7%, *P* < 0·0001). A relatively high proportion of residents with UI did not have any toileting programme in USA and Italy (22·8% and 12·3% respectively) whereas over 95% in Japan, Sweden and Iceland were on a toileting programme (*P*< 0·0001). Mean CMI ranged from the lowest 0·72 in Denmark to the highest in Sweden 1·02.	UI is highly prevalent in NH populations and significantly associated with increased age, being female, dependent mobility or being bedfast. Pads and briefs were the most common form of management across all countries with wide variation across countries in the use of scheduled toileting, toileting programmes or use of toilet facilities for residents with UI. There was also variation in the performance of testing for UTI and faecal impaction in residents with UI across countries. The term ‘nursing home’ is not a sound basis for cross-comparison due to differing cultures, policies and practices and use of resident-specific descriptors. Adjusting at the level of individual residents may be a better way forward. What was meant by scheduled toileting or toileting programmes was not described.	14/16 88%
**DuBeau 1999**, descriptive	USA; Data from NHs in 5 states: Kansas, Maine, Mississippi, New York and South Dakota	Investigate association between UI and QoL using MDS	133,111 eligible residents, mean age 84·2 years, sd 7. 79% (102,362) women; 21% men (30,749), 58,850 (65% with UI 2 times/week for last 3 months); mean age 84·3 years, sd 7·9, 78% (45,667) women; 22% (13,183) men	Quantitative. UI. Longitudinal cross-sectional retrospective documentary analysis using MDS for 6 months. UI, QoL using MDS Social Engagement Scale. ADL scale. Cognitive Performance Scale (CPS). MDS set 1994–1996.	68% (90,358) had consistent continence status with 44% (58,850) having UI 3 patterns of changes in continence status: decline (new or more severe UI), no change and improved. 68% evaluated for change in continence status. 83% unchanged, 12% declined, 5% improved. New or worsening UI associated with decline in QoL (anova: *F* = 391·6, *P*< 0·001). Significant association with worsening UI, decline in UI and worsening QoL over 6 months (OR: 1·46, 95% CI: 1·36–1·57). UI decline was second to the impact of cognitive decline (OR 2·06, 95% CI 1·93–2·21) and functional decline (OR: 1·78, 95% CI: 1·66–1·90) on worsening QoL.	UI is associated with a decline in QoL. There is a need to undertake interventions that improve or maintain continence.	20/20 100%
**Watson 2003**, descriptive	USA; non-random sample 52 NH in upstate New York (7458 beds)	Assess the use of the Agency for Health Research and Quality (AHRQ) guideline for UI (Fantl *et al.* 1996). Outcomes of new cases with UI following use of the guideline	200 randomly selected residents with new UI or newly admitted with UI over 12 weeks (103 newly admitted from potential 145/996 meeting criteria; 97 established residents with new UI from 171/6896 that met the criteria; UI 2 times/week for 4 weeks and stayed in 12 weeks). Mean age 82 years; sd 10·5, range 25–104. 69% (138) women; 31% (62) men. 96% Caucasian, 3% Black, 0·5% Hispanic, 0·5% Native American Reasons for not meeting criteria/loss provided and of potential 513 confirmed cases (259 newly admitted; 254 developed new UI); 11% died, terminal or hospice care 10%, discharge 9%, UI < 4 weeks 8% < paraplegic 0·2%	Quantitative. UI: defined. Retrospective chart review of cases identified by certified nursing assistants (CNA). The research team (3 advanced practice nurses gerontology and 2 physicians urology and gynaecology) checked the validity and judged as valid 70% of true cases (70% sensitivity) and 70% true non-cases (specificity) 4 weeks later by re-test eliminating 6% (35/548) as false. Reconfirmed by chart review of randomly selected cases for audit also excluded 6% (13/226) as false. Continence status, incidence of new UI, cognitive status (CPS). Ask CNA about management BT, PV, TV. 12-week period. Time 1 baseline, then time 2 4-week confirmation of UI status check, 1997–1998.	61% (122) had dementia; 51% (102) had severe or very severe cognitive impairment. 1·9% (145/7458) newly admitted with UI, 2·2% (171/7458) established residents developed new UI, so an incidence of 4·2% new UI in NH populations over a 12-week period. Rate of cases needing UI evaluation/assessment 1·9 new admissions per 100 beds and 2·3 established residents per 100 beds over 12 weeks. Only 15% (30/200) of cases had UI assessed by their clinician (doctor or nurse). Only 1 case had completely documented UI symptoms for the presence or absence of stress, urge or overflow UI. Documentation for the presence or absence of urge incontinence most common (15%), overflow (3%) and stress symptoms (3%). Frequency of episodes of UI (20%) and timing (2%). Expectations of families regarding UI management (2%). Frequency volume charts were non-existent. Fluid input and output records for 2 consecutive days, 47%. Rectal examination 15%, digital examination of prostate 1%, pelvic examination 2%, culture and sensitivity of urine sample 68%, urinalysis 56% and postvoid residual 6%. 81% had reversible cause of UI but only 34% had this addressed. 2% needed urological review. Only 3% received treatment 99% (197/200) used absorbent pads. 28% (56/200) had new UI management without cure as the aim. Routine scheduled toileting 80% (160/200): HT 14% (28/200), PV 15% (30/200). Overall 83% (166/200) had some form of toileting programme. Indwelling catheters 2% (4/200), intermittent catheterization 2% (3/200), penile sheaths 5% (3/62), pessaries for women 2% (1/138). All use of catheters was justified. The UI guideline had 90 standards that could be applied to each case; each case was assessed with the number of standards applicable to them (denominator) compared to the standards met (numerator). Compliance ranged from 0% to 45% with a mean compliance of 20% and a median of 21%. The number of standards for any single case ranged from 24 to 38 with a mean and median of 30. After 12 weeks, 6% (12/200) were continent. 4% (8/200) because of treatment of a reversible cause (mobility limitation, UTI, precipitating medication). 2% because of a toileting programme (scheduled toileting, prompted voiding) and 0·5% (1/2000) due to urethral dilatation for a urethral stricture. With 1 case attributed to precipitating.	Guideline has been underused and its use is feasible. The AHRQ guideline is generic and not specific to NH. Lack of awareness and familiarity with the guideline was identified as a barrier. Note: Cannot identify BT or TV with the scheduled toileting. BT not mentioned but may not be possible due to the high prevalence of cognitive impairment. Just TV but adding HT and PV numbers, add up to more than 200.	19/24 79%
**Jumadilova 2005**, descriptive	USA; 378 NH	Descriptive cross-sectional database analysis. Report of the prevalence of incontinence and treatment	All residents admitted in 378 NH between January 2002 and 31 December 2003. 29,645 eligible, mean age 78 years, sd 20–109. 63·8% (18,913) women; 36·2% (10,731) men; 89·2% White Caucasian. Mean LOS 116·3 days	Quantitative. MDS retrospective documentary analysis. Nursing progress notes, related care plans. 1 times MDS required annually, 2002–2003.	30% (8995) had some level of UI (0 = no incontinence 4 = incontinent most of the time). If those with FI were also included then UI prevalence was 58% (33,415 of 57,590). 8·7% of those with severe UI received drug treatment. Of those rated 1–4, only 8% received drug treatment Mean age increased with severity/level of UI as did LOS (*P*< 0·001). Residents with the more severe UI had more use of bladder training, use of incontinence pads and briefs and scheduled toileting (*P*< 0·001 for all).	UI and FI are prevalent. Drug treatment as a management modality was used in a minority. Bladder training, scheduled toileting and use of incontinence pads used to manage incontinence, and were associated with more severe incontinence. Mean age increased with the severity of incontinence. Note: What is meant by bladder training or scheduled toileting is not defined. Statistical tests for each result unclear	22/22 100%
**Wagg 2005**, descriptive	England, Wales, Northern Ireland Audit of GPs, acute hospital trusts and CHs. 100 CHs targeted and 309 randomized (but not specified) from 4 large groups, 85 (28%) agreed, only 29 (9% of total) provided data. 19 of the 56 that did not provide data due to lack of staff resources, annual or sick leave, staff or management changes (9), lack of or limited Internet access (3), difficulty with postal service (1)	Assess quality of care for people over 65 years for continence care following implementation of the NSF (DH 2001) that required integrated continence services by 2004	488 residents with UI mean age 86 years, sd = 8; 261 residents with FI mean age 84 years, sd = 8. Older than patients in hospitals or general population. Cognitive impairment 84%, functional impairment 75% (same for UI and FI samples)	Quantitative. Data collected for 20 residents with UI and 10 residents with FI or double incontinence for each CH and entered electronically into an audit data-collection schedule. Pilot work and main study demonstrated missing data were low. Reliability κ-values = 0·60 and above, so good agreement. Reliability checks made. Data collected on organizational structure and processes of care. Audit developed from national guidelines. Auditors were employees, 421 delegates attended workshops in 5 sites, 2004.	UI: 74% (20/27) stated integrated continence services were available locally. With 50% (10/20) stating that there was a lead person available and 13 WTE continence advisers (median 1·0, interquartile range 0·6–6·0) from the PCT and 8 WTE from the hospital (median 1·0, range 0–2·8). 100% CH routinely asked residents about bladder problems but did not necessarily follow through with an assessment. 86% (19/22) CH stated products supplied on clinical need not cost. 76% (19/25) sought patients’ views on the choice of products. Evidence of rationing in 19/25 CH. Median supply of products per day for each sector was 4. Documented continence history 70% (344/488) residents; nocturnal enuresis 43% (211/488); nocturnal frequency 33% (162/488); urinary frequency 32% (156/488); permanent catheter 13% (62/488). 89% (435/488) had a documented care plan. 82% (356) reviewed in the last 6 months, 41% (122/299) had a documented discussion about cause and treatment. 34% (115/341) bladder diary, evidence exacerbating medication reviewed/altered (29% 100/359); rectal examination 9% (43/488); urinalysis 65% (317); specialist examination 20% (100); of which documented evidence of abdominal examination for mass or retention (77%, 77), perineum and pelvis, prolapse, pelvic floor contraction, atrophy (44%, 44), rectal examination (25%, 25); Post residual volume 20% by ultrasound or catheter (90/448); clear type of UI 40% (166/418), specific treatment plan 82% (400/488); advice on general health 25% (122/488); advice on lifestyle 16% (79/488); behaviour modification 6% (29/488); bladder training 16% (80/488); containment 63% (307/488); management of faecal impaction 16% (80/488); oestrogen treatment 0·4% (2); PFME 3% (15/488); pharmacological interventions 14% (68/488); surgery 1% (5/488); toileting schedules 60% (291/488); treatment of comorbidities 10% (48/488); treat UTI 22%(105/488); other 6% (29); none of the above or other 6% (29/488). FI or mixed: 27 CHs. 74% (20) CHs integrated continence service available; lead person 50% (10); access to continence specialist 96% (26/27); 100% CH routinely asked about bowel problems; 96% had access to specialist continence advice (median 1·0).; privacy and dignity reported as being maintained by 100% CH; written policy 93% (25/27); integrated care pathway 12% (3/26); written protocol for assessment 88% (23/26); documented history 50% (range 45–63%). Structured programme for continence for staff 63% (17/27); includes basic assessment 65% (11/17); specialist continence assessment conducted by practitioner 41% (11/27); areas for assessment and treatment maintain privacy and dignity 100% 27/27; bladder ad bowel care subject to regular audit 64% 14/22; evidence-based information freely available to patients and carers 85% (23/27). Management plans available in 76% (20) CHs. Documented treatment plan 76% (198/261); treatment goals recorded 54% (74/138); advice on general health 12% (32); advice on lifestyle 9% (23); colostomy or ileostomy 2% (5); FI chart 33% (87); bowel training/regimes techniques 13% (33); improved mobility 17% (44); improved quality of access to toilet facilities 14% (36/261); Pelvic floor training 1% (3); pharmacological interventions 27% (70); medications review 29% (76); surgery 2% (5); toileting schedules (52% (137); treatment of comorbidities 8% (22); others 15% (39); none documented 15% (38).	Requirements for integrated continence services not yet met. Urgent need to establish fundamentals of continence care in medical and nursing practice Findings specific to CHs: based on data extraction was better than expected. Majority had documented care plans; less than half and, in some cases, a minority had active management of UI and FI No evidence of continence being maintained due to focus of audit being the management of UI and FI.	22/22 100%
**Schnelle 1988**, descriptive/economic analysis	USA, 3 NH	Assess time used assisting residents in toileting, changing incontinent residents and identify associated costs	231 residents all aged over 65 years UI assessed. 242 included in the study who were able to consent	Quantitative. UI: defined. 21-item toileting schedule (independent toileting inventory). No blinding. Direct time observations of changes and toileting over a 9-month period. Initially 14 days of hourly checks. Time observations over 9 months. Reliability checks by 2 independent observers of same staff (93% agreement).	51% (123) UI and 49% (119) were continent. Of those with UI, 8·9% (11) could toilet independently, 13% (16) were partially dependent, 43·1% (53) dependent for some assistance, 35% (43) were dependent = 78% (96) were dependent on assistance for toileting. Residents who were continent were more capable of independent toileting compared to those with UI (81·5%, 97% vs. 8·9%, 11, χ^2^ = 190·5, *P*< 0·0001). Based on 116 cleaning episodes and 132 toileting episodes, toileting required more time (mean: 7·97 vs. 5·55 minutes, *t* = 2·97, *P*< 0·004). Total time to toilet a resident was a mean of 2·42 minutes more than the cleaning time due to travel time and assistance.	Residents with UI who are physically dependent are incapable of independent toileting. The time taken to toilet exceeds cleaning residents. It takes more time to maintain continence in a dependent resident than to manage incontinence.	10/28 36%
**Ouslander 1984**, descriptive/economic data	USA, 7 NH	Identify and estimate costs of managing UI in NHs	All residents with UI with or without concomitant *F*. *n* = 419 in NHs	Quantitative. SUI: not defined in the paper. Survey of staff, administrators, nursing staff and aides. Data from medical supply companies and laundry company. Modelled costs of incontinence for first daily management (supplies, laundry, labour) and second-order costs (managing complications). Data on labour costs. Data on management techniques: disposable bedpads, reusable bed and incontinence pads, disposable incontinence pads and indwelling catheters. Questionnaire about knowledge and attitudes of staff, 1982.	First-order costs of the 4 common methods of managing UI range between $2·9 and $11·09 per incontinent patient/day. Estimated costs of UI using 419 residents in 7 NHs between $0·5 and $1·5 billion (3–8%) of costs of NH care. Management of UI with indwelling catheters results in lowest first-order costs with the highest for residents managed by disposable bed pads.	More active assessment and treatment of UI could result in considerable cost savings	11/26 42%

UI, urinary incontinence; FI, faecal incontinence; OR, odds ratio; CH, care home; LOS, length of stay; NH, nursing home; PCT, primary care trust; QoL, quality of life; WTE, whole time equivalent.

### Data synthesis

The studies differed in their aims, methods, outcome measures, patient characteristics and quality. The review adopted a broad approach to capturing relevant empirical studies to encapsulate and describe the extent of work related to management of incontinence in care homes and was not restricted to a narrow focus. Narrative synthesis using techniques from metastudy for primary qualitative research provided summary description for all included studies and allowed contrast and comparison of quantitative and qualitative data (Paterson *et al.* 2001).

## Results

### Descriptive observational studies

Twenty three descriptive or observational studies were located, of which 10 studies had UI as the primary focus (Table 3). These studies included cohort, case study or survey design related to UI as the primary outcome either with or without concomitant FI (*n* = 8; Ouslander 1982, Palmer 1991, Peet 1996, Sgadari 1997, DuBeau 1999, Watson 2003, Jumadilova 2005, Wagg 2005) or economic evaluation of managing UI (*n* = 2; Ouslander 1984, Schnelle 1988) (Table 3). Studies associated with other conditions or management approaches, with UI not the primary focus are not reported in this study (*n* = 13).

### Dates of publication and data collection of descriptive studies with management of urinary incontinence as primary focus

Included studies spanned the period 1980 to 2005 (Ouslander 1982, 1984, Schnelle 1988, Palmer 1991, Peet 1996, Sgadari 1997, DuBeau 1999, Watson 2003, Jumadilova 2005, Wagg 2005). Four descriptive studies did not include dates of data collection (Ouslander 1982, Schnelle 1988, Sgadari 1997, Wagg 2005).

#### Countries and settings

Most studies were undertaken in the USA (seven) with two in England (Peet 1996) or England, Wales and Northern Ireland (Wagg 2005), and one international study involving seven countries (Sgadari 1997). The majority of studies (*n* = 8), where stated, included a total of 542 care homes (ranging from 3 per study to 378 care homes per study). Two studies did not specify how many homes were included (Sgadari 1997, DuBeau 1999). Eight studies with UI as the primary focus are reported followed by the two studies that focused on the economic evaluation of management of UI.

### Studies with management of urinary incontinence as primary focus

Seven studies comprised retrospective documentary review at the level of care home and/or residents at one or a number of time points and one study formed part of a national audit (Wagg 2005) (Table 3). Inclusion criteria were specified in all eight studies but varied between the individual studies and were not comparable. Exclusion criteria also varied between the studies and were not comparable and were not specified in two studies (Sgadari 1997, Wagg 2005).

#### Participants/samples

Seven studies specified diagnostic groups and varied across the studies in relation to UI and FI and comorbidities (Ouslander 1982, Palmer 1991, Sgadari 1997, DuBeau 1999, Watson 2003, Jumadilova 2005, Wagg 2005). Mean age or gender were not included in two studies (Ouslander 1982, Sgadari 1997), although, in the latter study, age ranges were provided and 40% (111,676) of the sample were over 85 years of age, with a majority of the samples women (range 65·9–76·2% across seven countries). Of those studies that included mean age and gender participants had a mean age of 82·5 years (*n* = 164,235; Palmer 1991, Peet 1996, DuBeau 1999, Watson 2003, Jumadilova 2005, Wagg 2005). Two studies specified ethnic origin with a majority of residents being White (89·2%, Jumadilova 2005; 96%, Watson 2003).

#### Methods

The main methods involved documentary review and analysis (medical records or case notes) at one time point (Ouslander 1982, Peet 1996, Sgadari 1997, Jumadilova 2005, Wagg 2005) with follow-up review at one (Watson 2003), two (DuBeau 1999) or three time points (Palmer 1991). UI was verified by researchers or individual assessment (Palmer 1991, Peet 1996, Watson 2003) or by the analysis of a cross-sectional database of 6 months or 2 years of newly admitted residents (Jumadilova 2005). All studies involved the collection of quantitative data from documentary evidence, which included data on UI, FI and comorbidities, mental status, functional dependence, activities of living and continence management.

Power calculations or an indication of the sample size required was not specified in seven studies, while Wagg (2005) provided an estimate. Loss to follow-up was included in two studies (Palmer 1991, Watson 2003).

#### Prevalence and incidence

Seven studies included prevalence of UI with two studies also including incidence (Palmer 1991, Watson 2003). A prevalence estimate was not possible in the national audit (Wagg 2005). The definition of UI was not specified in all studies and varied between those that did (Ouslander 1982, Sgadari 1997, DuBeau 1999, Watson 2003, Jumadilova 2005). Prevalence of UI ranged from 30% to 65% (Sgadari 1997, Jumadilova 2005), FI 22·4% to 55·5% (Sgadari 1997) and both UI and FI from 20·5% to 64% (Ouslander 1982, Sgadari 1997) between the studies and care home populations (Table 3). Prevalence in women was reported as being higher than in men (77·6% of 58,850 that represented a 65% prevalence rate, DuBeau 1999). One study reported that 69% of care home residents had UI most days and, for 39%, it was severe (Peet 1996). Jumadilova (2005) found 30% of residents had some level of UI. Looking at change in prevalence over time Palmer (1991) found 39% with UI at 2 weeks, 37% at 2 months and 44% at 1 year (of whom 90% had dementia). DuBeau (1999) estimated a change in UI for 68% of the sample over 6 months. Change was defined as decline (new or more severe UI), no change or improved. They found 83% were unchanged, 12% declined and 5% improved with a statistically significant association found between UI decline and worsening UI with worse quality of life (QoL; *P*< 0·00001).

For the two studies that included an estimate of incidence of UI, one reported a rate of new cases over 1 year as 27% with men having a higher incidence of UI than women (9/16 vs. 21/96, *P*< 0·004). Watson (2003) found 1·9% of newly admitted residents had UI whereas 2·2% of established residents developed new UI over a 3-month period.

#### Urinary incontinence and associated factors

The studies found UI associated with a number of factors and comorbidities. Ouslander (1982) found people with UI had cognitive impairment, limitations with mobility, skin irritations or urinary tract infection (UTI). They also had one or two related comorbidities, dementia (45%), stroke (28%), Parkinson disease (9%), depression (6%), paraplegia, tumours and bladder cancer (<5%). Peet (1996) reported the majority of people with UI had problems with mobility (85%). UI was significantly associated with having dementia, dependent walking and dependent transfer at 2 weeks (all, *P*< 0·001; Palmer 1991). UI at 2 weeks and dementia at 2 months were also significantly associated with UI at 1 year (*P*< 0·05). UI increased significantly over 1 year (*P*< 0·001). However, age was not associated with incidence or prevalence. Dementia was highly prevalent in new cases of UI (90%). One-year risk factors were calculated using multiple regression analysis, the highest factor being male (68%), presence of UI at 2 weeks, poor behavioural adjustment to the care home at 2 weeks and dementia at 2 months. Patients with these attributes had a cumulative increased risk factor of UI at 122%. Patients with no detectable mental morbidity had the lowest incidence of UI. FI was associated with UI (*P*< 0·05). Resolution of UI was significantly associated with the ability to ambulate, transfer independently, absence of FI and dementia (*P*< 0·05; Palmer 1991).

In the study across seven countries (Sgadari 1997), positive associations between age and UI were found but not in Sweden and Iceland. Dependent locomotion was significantly associated with UI in all countries (*P*< 0·001) and cognitive status, with mild-to-severe impairment associated with UI (*P*< 0·001). UTI was only associated with UI in four countries (*P*< 0·001). This study demonstrates that cultural variations should be allowed for and findings between countries cannot be generalized. However, impairment in cognition and mobility are significantly associated with UI and UI is highly prevalent in care home populations.

These findings are supported by DuBeau (1999) who found a statistically significant association between new or worsening/decline in UI and lower QoL, and improving UI with better QoL (*P*< 0·001). There was a statistically significant association between lower QoL and UI in people with moderate activity of daily living (ADL) impairment regardless of cognitive status (*P*< 0·001). Multivariate analysis demonstrated UI was associated with a decline in QoL (OR = 1·46, 95% CI: 1·36–1·57), second to the impact of cognitive decline (OR = 2·06, 95% CI: 1·93–2·21) and functional decline (OR = 1·78, 95% CI: 1·66–1·90) on worsening QoL. Jumadilova *et al.* (2005) found mean age and length of stay increased with severity of UI (*P*< 0·001).Only four studies included assurance of the reliability of the data collected in their report of methods (Palmer 1991, DuBeau 1999, Watson 2003, Wagg 2005).

#### Results related to management of incontinence

##### Assessment and documentation

Both Ouslander (1982) and Watson (2003) reported that <15% of residents had their incontinence recorded in care home records or efforts made to evaluate it. Watson (2003) found only 15% of cases had their UI assessed by their clinician (doctor or nurse) and only one case had completely documented UI symptoms for the presence or absence of stress, urge or overflow. Documentation for the presence or absence of urge UI was most common (15%), but only for the minority. Frequency of episodes of UI (20%) and timing (2%) were also only recorded for a minority. Frequency volume charts of UI were non-existent, although fluid input and output records for two consecutive days were available for nearly half of residents (47%). Clinical examinations were undertaken for a minority. 81% had a reversible cause of UI at onset but only 34% had this addressed and 3% received treatment (*n* = 6).

More recent data from a national audit in care homes in England, Wales and Northern Ireland (Wagg 2005) that reviewed the management of UI found 74% of care homes stated integrated continence services were available locally, with 50% stating there was a lead person available. All care homes routinely asked residents about bladder problems but did not necessarily follow through with an assessment. A documented continence history was available for 70% of residents. 89% of residents had a documented care plan, 82% were reviewed in the last 6 months, 41% had a documented discussion about cause of UI and treatment with 34% having a bladder diary or frequency volume chart. A clear type of UI was recorded for 40% of residents, and a specific treatment plan for the majority (82%). The audit also investigated the management of FI or mixed FI and UI as a separate entity Documented treatment plans for 76% of residents reviewed were available, with treatment goals recorded for 54%, advice on general health (12%), advice on lifestyle (9%), FI chart (33%) and documented history available (50%; range 45–63%).

##### Policies

Peet (1996) found that most care homes had policies for managing incontinence (use of pads 87%, use of continence sheets 62%, personalized bathing policy 51%, use of appliances or catheters 41%, with 21% having a policy on intake of incontinent residents who were precluded from admission) and the promotion of continence using a variety of strategies (day time toileting 83%, use of aids – 68%, night toileting 52%, use of toilet signs 49% with only 38% restricting or adjusting fluids.

The national audit that investigated the management of UI and FI or mixed FI and UI as a separate entity (Wagg 2005) found 96% of care homes had access to a continence specialist (median 1·0), 100% of care homes routinely asked about bowel problems, with privacy and dignity reported as being maintained by 100% of care homes and having a written policy (93%), integrated care pathway (12%) or written protocol for assessment (88%). Structured programmes on incontinence for staff were available in 63% of homes and included basic assessment (65%), specialist continence assessment conducted by practitioner (41%), areas for assessment and treatment to maintain privacy and dignity (100%), with bladder and bowel care subject to regular audit (64%) and evidence based information freely available to patients and carers (85%). Management plans were available in 76% of care homes. Eighty-six per cent of care homes stated products were supplied on clinical need and not cost and 76% sought patients’ views on choice of products. There was evidence of rationing in 76% of care homes and the median supply of products per day was four.

##### Management techniques

Peet (1996) reported on aids used, continence promotion and aspects of management requiring change. Continence promotion techniques for 57% of residents included [toileting 46%, fluid adjustment 16%, bladder training (BT) 6%]. More than half of residents received one or more techniques for the management of UI or promotion of continence (57%), although pelvic floor muscle exercises had not been undertaken with any residents. Based on the assessment of individual cases (*n* = 96), it was judged that their management of UI should be changed for some residents in relation to use of pads (25%), drug therapy (15%) appliances (9%), investigations (47%), BT (31%), toileting (27%), use of continence aids (12%) or fluid intake (9%). Fifty-seven per cent of homes (*n* = 39) requested more support from a continence service.

Care practices for managing UI in individual cases varied across countries with the testing of incontinent residents for UTI ranging from 48% in France to 11% Italy and Sweden and faecal impaction ranging from 0·5% in Sweden to 27·3% in France. Use of scheduled toileting ranged from 50·7% in Iceland to 5·6% in Italy and the use of pads for individual management ranged from 71·6% in the USA to 92·9% in Iceland. Use of pads was the most common management technique used for managing UI in care homes. Having no toileting programmes or use of the toilet for people with UI ranged from 22·8% in the USA to 2·6% in Italy and 52·7% in Japan to 4·4% in France. Use of pads was the most common management strategy and there was marked variation in the use of scheduled toileting programmes, and it was not apparent what these involved (Sgadari 1997).

A more recent study showed that expectations of families with regard to UI management were recorded in only 2% of instances. They also found appropriateness of treatment against the Agency for Health Research and Quality (AHRQ) guideline (Fantl 1996) could not be evaluated in all cases because of a lack of an UI diagnosis (Watson 2003). In relation to UI management, a majority 99% used absorbent products, and 28% had new UI management but without cure as the aim. This involved routine scheduled toileting; timed voiding (80%), habit retraining (14%) or prompted voiding (15%). Overall, 83% had some toileting programme. It is not clear if BT was used as part of scheduled toileting or just timed voiding as it was not mentioned, although BT may not be suitable due to the high prevalence of cognitive impairment in residents. Indwelling catheters (IC)(2%), intermittent catheterization (2%), external sheaths for men (5%) and pessaries for women (2%) were used by a minority. All use of catheters was justified.

The AHRQ UI guideline (Fantl *et al.* 1996) had 90 standards that could be applied and each case was assessed with the number of standards applicable to them. Compliance ranged from 0% to 45% with a mean compliance of 20% and a median of 21%. After 12 weeks, 6% of residents were continent; 4% because of treatment of a reversible cause (mobility limitation, UTI, precipitating medication) and 2% because of a toileting programme (scheduled toileting, prompted voiding). Watson (2003) concluded the guideline had been underused but its use was feasible. The AHRQ guideline is generic and not specific to care homes. Staff awareness and familiarity with the guideline was identified as a barrier.

Jumadilova (2005) found only 8% of residents with UI were treated with drugs, with 8·7% of those with severe incontinence receiving drugs. A statistically significant association was found with more severe UI and the use of BT (*P*< 0·001), incontinence pads/briefs (*P*< 0·001) or scheduled toileting (*P*< 0·001).

The audit of care homes found documented treatment plans for 76% of residents were available. They included: treatment goals recorded (54%), advice on general health (12%) or lifestyle (9%), an FI chart (33%), bowel training/regimes techniques (13%), improved mobility (17%), improved quality of access to toilet facilities (14%), pelvic floor muscle exercises (1%), drug therapy (27%), medications review (29%), surgery (2%), use of toileting schedules (52%), treatment of comorbidities (8%), other interventions or none of these documented (15%) (Wagg 2005). The audit findings indicate that practice in care homes has developed as the studies from the 1980s, early 1990s and that the majority of residents had documented care plans, although less than half and, in some cases, a small minority active management of UI and FI.

In all of the above studies, no economic data regarding the management of UI and outcomes were included. The quality ratings of the studies ranged from 54% to 100%, with two studies achieving the maximum score (Jumadilova 2005, Wagg 2005).

Details of research funding were included in a minority of studies (Palmer 1991, Watson 2003, Jumadilova 2005, Wagg 2005).

### Studies with an economic evaluation of managing urinary incontinence

The focus of two studies was economic evaluation of managing incontinence based on observational and descriptive data (Ouslander 1984, Schnelle 1988). Estimates of costs of management were calculated from survey data from staff questionnaires from 16 care homes (Ouslander 1984, including data from seven nursing homes from a previous study, Ouslander 1982). Data were based on patients with UI, with or without FI, but sample size and ages were not included nor the response rate for staff questionnaires returned which the data were based on. Schnelle (1988) based estimates on 231 incontinent and continent residents who consented (92%, *n* = 252) from three nursing homes (extending data from Ouslander 1982). All residents were stated to be aged 65 years and above but details on age, gender, and ethnicity were not reported in either study and no exclusion criteria were specified. Only continence status was stated with no other diagnostic conditions or comorbidities reported.

#### Methods

Cost estimates were derived from staff questionnaire surveys, medical supply companies and a laundry company for incontinence products (Ouslander 1984) and by direct observation of care for toileting and cleaning (Schnelle 1988). Models of costs were developed based on supplies, laundry and labour (first-level costs) and complications (second-level costs related to skin or treatment of UTI in the home or hospital) (Ouslander 1984) and staff time and laundry (Schnelle 1988). Dates of data collection were not specified, although Schnelle (1988) obtained data over 9 months using a 21-item toilet assessment inventory (with 93% agreement) while the Ouslander's (1984) study was from one time point. No indication of sample size or power calculation were included. Quality ratings were 36% and 42%.

#### Prevalence and incidence

Prevalence of UI was not reported by Ouslander (1984), although Schnelle (1988) stated 51% of residents had UI and 49% were continent in the nursing homes studied.

#### Outcomes for management of incontinence

Ouslander (1984) reported a trend for lowest costs being incurred for residents managed with indwelling urinary catheters (range: $2·90 minimum of 1 bed change per day–$5·11 maximum of 5 bed changes per day) vs. the highest with disposable bed pads (range $6·91 minimum 3 bed changes per day –$11·09 maximum bed changes per day) based on first-order costs. If second-order costs were estimated based on the incidence of complications, yearly costs per patient with use of a catheter were $2888 compared to the cost of a patient without a catheter from $2072 to $4532. They concluded the use of indwelling catheters for the management of UI was not justified.

Schnelle (1988) reported 78% of residents with UI were dependent for toileting vs. 7% who were continent. Only 9% of residents with UI were able to toilet independently compared to 82% who were continent (*P*< 0·0001). Based on observation of 116 cleaning and 132 toileting care episodes, the total time to toilet a resident was 2·42 minutes more than the cleaning time (7·97 vs. 5·55 minutes, *P*< 0·004) due to the additional time of travel and assistance. They concluded that it costs more to maintain continence in a dependent resident than it takes to manage incontinence, whereas Ouslander (1984) concluded that active evaluation and treatment of UI could provide cost savings and improve well-being of patients and carers. A comprehensive economic evaluation of maintaining continence and managing incontinence in care home populations is warranted. The quality rating of both studies was low, 36% and 42%. Neither study specified their source of funding.

## Discussion

This review found that descriptive studies for the management of incontinence and promotion of continence in care homes involved mainly women with a mean age above 80 years, which reflects prevalence of the condition and care home populations. The prevalence of UI was higher than FI with more women affected than men, which is in keeping with findings of community populations with the range of prevalence higher in these institutional settings ((McGrother *et al.* 2003, Fonda *et al.* 2005). No studies in the review were aimed at maintaining continence in residents in care homes.

### Methodological issues and limitations

The studies were restricted to only those published in English. They comprised mainly documentary analysis and review and may have limited reliability and validity. Only three studies followed up residents over time (Palmer 1991, Peet 1996, Watson 2003), with Palmer (1991) following up to 1 year. Quantitative data were collected with only four studies reporting on the reliability of data (Palmer 1991, DuBeau 1999, Watson 2003, Wagg 2005). Power calculations to justify sample sizes and loss to follow-up did not generally feature. The overall quality of studies was variable with the economic evaluations scoring the lowest total scores, which may reflect the focus of the reports and limited methodological information included.

### Populations

The range of data collected included UI and FI status, mental status, functional abilities, ADLs and comorbidity. The severity of incontinence was not routinely recorded. The majority of residents with UI had problems with mobility and/or dementia and were found to be significant (Palmer 1991) with deterioration in UI significantly affecting QoL (DuBeau 1999), which indicates interventions aimed at improving or preventing incontinence are warranted. The one international study reported variations across care home populations for prevalence of incontinence and approaches to management between countries (Sgadari 1997), which reflect organizational, cultural, management, policy and practice differences in this care sector within countries making comparison difficult. Studies targeted in care homes within countries are warranted.

Studies from the 1980s and 1990s generally found that incontinence had not been documented or assessed with the exception of the study reported in 2003 (Watson 2003) who compared documentation and management with the AHRQ guidelines (Fantl *et al.* 1996) and who also reported only a minority of residents having their incontinence documented or assessed. A more recent national audit of care homes, however, reported that a majority of 70% did have a history of incontinence recorded, with 89% having a documented care plan and 82% or residents having been reviewed in the previous 6 months (Wagg 2005). These changes in practice reflect recognition of the need to manage incontinence and promote continence, available research evidence as well as the development and implementation of guidelines within countries (Fantl *et al.* 1996, Button *et al.* 1998, NICE 2006, 2007, Abrams *et al.* 2009), although none were specifically developed for care home populations.

### Policies and management

Use of incontinence pads and toileting were the most prevalent forms of management and feature of documented policies. The detail on toileting programmes used was not explicitly specified but stated to include BT, scheduled toileting and prompted voiding. As has been previously reported, operational definitions and content of toileting programmes have not been included in studies and their theoretical basis requires revisiting (Roe *et al.* 2007a,b;) so they reflect contemporary developments in behavioural techniques and interventions. Habit retraining, timed and prompted voiding are common toileting practices used in care home populations for residents with cognitive or physical impairments, with limited evidence on effectiveness for timed voiding (Eustice *et al.* 2000, Ostaszkiewicz *et al.* 2004a,b;). Recent studies have included prompted voiding with physical exercise for residents with some evidence of effectiveness (Schnelle *et al.* 2002) and reflect the combined interventions and approaches being adopted for managing incontinence (Roe *et al.* 2007a). Other forms of management, such as, pelvic floor muscle exercises, drugs, catheters or penile sheaths featured less frequently. Wagg (2005) found treatment goals were documented for 54% of residents with 76% of homes reporting that they would involve residents in choice of incontinence products. However, only 2% of family members were reported as being involved in decisions on the management of incontinence by Watson (2003). No studies reported involving residents in decision-making regarding their management goals.

Two studies assessed whether the current management of incontinence should be changed and identified this was the case for a minority of residents (Peet 1996, Watson 2003). Watson (2003) concluded that the AHRQ guideline (Fantl *et al.* 1996) had been under utilized in care homes but its use was feasible. These studies spanning the last 30 years demonstrate that there are improvements in the implementation of care for managing incontinence in care home residents. However, there is a lack of longitudinal studies incorporating documentary review and observed practice for these populations and further research is warranted to determine outcomes and improvements in continence status. Studies targeted at maintaining continence in residents who are continent should also be undertaken.

### Economic evaluation

Only two studies gave an economic evaluation (Ouslander 1984, Schnelle 1988). Costs of using indwelling catheters were more expensive than using incontinence pads (Ouslander 1984), whereas toileting residents incurred more costs due to the increased time required than changing and cleaning them (Schnelle 1988). Schnelle (1988) concluded that it costs more to maintain continence in dependent residents than managing incontinence. Economic evaluations of maintaining continence and different interventions for managing incontinence are justified.

## Conclusion

Combined evidence suggests that conservative approaches for managing incontinence and promoting continence involving pads and toileting are most frequently used for residents in care homes. Improvements in documenting practice and assessment of incontinence have been identified over the last three decades, although there are variations between and within countries. Involving residents or family members in decisions for managing incontinence is poorly reported and should be more widely practised. Studies on maintaining continence and identification of components of toileting programmes that make them successful incorporating full economic evaluation are warranted.

**What is already known about this topic**

- Incontinence is a prevalent condition among older people living in care homes with reported range of prevalence variable across studies and populations.
- Incontinence incurs personal and institutional costs in terms of quality of life, staff time, laundry, use of aids and appliances.
- Research on the management of incontinence in care home populations has been undertaken predominantly in the USA by designated research teams and findings may not be transferable to other populations or settings where the organization, staffing and delivery of care may vary.

**What this paper adds**

- There is emerging evidence that the management of incontinence and promotion of continence is an increasing feature of practice within care homes reflected by the increased availability of policies and documented care.
- Use of incontinence pads and toileting regimens are the most common forms of care for older people with incontinence in care home populations.
- The evidence base, theories underpinning toileting practices or their form, frequency and content are unclear from the studies reviewed but are stated to comprise bladder training, scheduled or prompted voiding.

**Implications for practice and/or policy**

- Involving residents of care homes or family members in decisions for managing incontinence should be more widely practised.
- Studies on maintaining continence and identification of components of toileting programmes that make them successful along with economic evaluation are warranted.

## Supporting Information

**Table S1.** Studies awaiting assessment.

**Table S2.** List of excluded studies.

## Notes

The *Journal of Advanced Nursing (JAN)* is an international, peer-reviewed, scientific journal. *JAN* contributes to the advancement of evidence-based nursing, midwifery and health care by disseminating high quality research and scholarship of contemporary relevance and with potential to advance knowledge for practice, education, management or policy. *JAN* publishes research reviews, original research reports and methodological and theoretical papers.

For further information, please visit *JAN* on the Wiley Online Library website: http://www.wileyonlinelibrary.com/journal/jan

**Reasons to publish your work in *JAN*:**

- **High-impact forum:** the world's most cited nursing journal and with an Impact Factor of 1·518 – ranked 9th of 70 in the 2010 Thomson Reuters Journal Citation Report (Social Science – Nursing). *JAN* has been in the top ten every year for a decade.
- **Most read nursing journal in the world:** over 3 million articles downloaded online per year and accessible in over 7,000 libraries worldwide (including over 4,000 in developing countries with free or low cost access).
- **Fast and easy online submission:** online submission at http://mc.manuscriptcentral.com/jan.
- **Positive publishing experience:** rapid double-blind peer review with constructive feedback.
- **Early View:** rapid *online* publication (with doi for referencing) for accepted articles in final form, and fully citable.
- **Faster print publication than most competitor journals:** as quickly as four months after acceptance, rarely longer than seven months.
- **Online Open:** the option to pay to make your article freely and openly accessible to non-subscribers upon publication on Wiley Online Library, as well as the option to deposit the article in your own or your funding agency's preferred archive (e.g. PubMed).
