# Supplementary material for: Systematic review of the management of incontinence and promotion of continence in older people in care homes: descriptive studies with urinary incontinence as primary focus
Source: J Adv Nurs. 2011 Feb;67(2):228–50. doi: 10.1111/j.1365-2648.2010.05481.x (PMC3132440; doi:10.1111/j.1365-2648.2010.05481.x)
Supplement: Supplementary file 1 [file jan0067-0228-SD1.doc]

AWB Table A1 Studies Awaiting Assessment

| Al-Samarrai NR, Uman GC, Al-Samarrai T, Alessi CA (2007) Introducing a new incontinence management system for nursing home residents*. Journal of the American Medical Directors’ Association* 8, 4, 253-261.  Aslan E, Komurcu N, Beji NK, Yalcin O (2008) Bladder training and Kegel exercises for women with urinary complaints living in a rest home. *Gerontology* 54, 4, 224-231.  Bliss DZ, Zehrer C, Savik K, Smith G, Heblom E (2007) An economic evaluation of four skin damage prevention regimens in nursing home residents with incontinence: economics of skin damage prevention. *Journal of Wound, Ostomy and Continence Nursing* 34, 2, 143-152.  Fink HA, Taylor BC, Tacklind JW, Rutks IR, Wilt TJ (2008) Treatment interventions in nursing home residents with urinary incontinence: a systematic review of randomized trials. *Mayo Clinic Proceedings* 83, 12, 1332-1343.  Levy-Storms L, Schnelle JF, Simmons SF (2007) What do family members notice following an intervention to improve mobility and incontinence care for nursing home residents? An analysis of open-ended comments. *The Gerontologist* 47,1, 14-20.  MacDonald CD, Butler L (2007) Silent no more: elderly women’s stories of living with urinary incontinence in long-term care *Journal of Gerontological Nursing* 33,1, 14-20.  O’Dell KK, Jacelon C, Morse AN (2008) ‘I’d rather just go on as I am’ – Pelvic floor care preferences of frail., elderly women in residential care. *Urologic Nursing* 28, 1, 36- 47.  Rodriguez NA, Sackley CM, Badger FJ (2007) Exploring facets of continence care: a continence survey of care homes for older people in Birmingham*. Journal of Clinical Nursing* 16, 5, 954-962.  Sackley CM, Rodriguez NA, van den Berg M, Badger F, Wright C, Besemer J, van Reeuwijk KT, van Wely L (2008) A phase II exploratory cluster randomized controlled trail of a group mobility training and staff education intervention to promote urinary continence in UK care homes. *Clinical Rehabilitation* 22, 8, 714-721.  Saxer S, de Bie RA, Dassen T, Halfens RJ (2008) Nurses’ knowledge and practice about urinary incontinence in nursing home care*. Nurse Education Today*. 28, 8, 926-934.  Shamiliyan T, Wyman J, Bliss DZ, Kane RL, Wilt TJ (2007) Prevention of urinary and fecal incontinence in adults. *Evidence Report Technology Assessment (Full Report)* 16, 1, 1-379.  Tanaka Y, Nagata K, Tanaka T, Kuwano K, Endo H, Otani T, Nakazaa M, Koyama H (2009) Can an individualized and comprehensive care strategy improve urinary incontinence (UI) among nursing home residents? *Archives of Gerontology and Geriatrics* 49, 2, 278-283.  Van Houten P, Achterberg W, Ribbe M (2007) Urinary incontinence in disabled elderly women: a randomized clinical trial on the effect of training mobility and toileting skills to achieve independent toileting*. Gerontology* 53, 4, 205-210.  Wagg A, Lowe D, Peel P, Potter J (2008) Continence care for older people in England and Wales: data from a national audit. *Journal of Wound, Ostomy and Continence Nursing* 35, 2, 215-220.  Wai AA, Fook VF, Jayachandran M, Biswas J, Nugent C, Mulvenna M, Lee JE, Kiat PY (2008) Smart wireless continence management system for persons with dementia. *Telemedicine Journal and E Health* 14, 8, 825-832. |
| --- |
