# Supplementary material for: Systematic review of the management of incontinence and promotion of continence in older people in care homes: descriptive studies with urinary incontinence as primary focus
Source: J Adv Nurs. 2011 Feb;67(2):228–50. doi: 10.1111/j.1365-2648.2010.05481.x (PMC3132440; doi:10.1111/j.1365-2648.2010.05481.x)
Supplement: Supplementary file 2 [file jan0067-0228-SD2.doc]

Accessory Web-Based -Table 2. List of Excluded Studies

| Adelmann PK. (2004) Prevalence and detection of urinary incontinence among older medicaid recipients. *Journal of Health Care for the Poor and Underserved,* **15**, (1), 99-112.  Al - Tvreihi F.I., Hasson A., Wolf-Klein G. & Isenberg M. (2005) Albumin, length of stay and proton pump inhibitors: key factors in C. difficle associated disease in nursing home patients. *Journal of the American Medical Directors Association,* **6**,(2), 105-108.  Alessi C.A., Schnelle J.F., Traub S. & Ouslander J.G. (1995) Psychotropic medications in incontinent nursing home residents: association with sleep and bed mobility*. Journal of the American Geriatrics Society,* **43**,(7), 788-792.  Al-Qutob R. (2001) Menopause-associated problems: types and magnitude. A study in the Ain Al-Basha area, Jordan. *Journal of Advanced Nursing,* **33**,(5), 613-620.  Ang Y.H., Au S.Y.L., Yap, L.K.P. & Eo, C.H. (2006) Functional decline of the elderly in a nursing home. *Singapore Medical Journal,* **47**, (3), 219-224.  Bates-Jensen B.M., Cadogan M., Osterweil D., Levy-Storms L., Jorge J., Al-Samarrai N., Grbic V. & Schnelle J.F. (2003) The Minimum Data Set Pressure Ulcer indicator: does it reflect differences in care processes related to pressure ulcer prevention and treatment in nursing homes? *Journal of the American Geriatrics Society,* **51,** (9), 1203-1212.  Baumgarten M., Margolis D., Van Doorn C., Baldini A.L., Hebel J.R., Zimmerman S. & Magaziner J. (2004) Black/White differences in pressure ulcer incidence in nursing home residents. *Journal of the American Geriatrics Society,* **52**,(8), 1293-1298.  Bellar A. (2002). The influence of environment on resistiveness to care and the effectiveness of an intervention to decrease resistiveness to care in people with Alzhemer's disease residing in institutions. Unpublished PhD Thesis. Wayne State University.  Binder E.F., Kruse R.L., Sherman A.K., Marsden R., Zweig S.C., D'Agostino R. & Mehr D.R. (2003) Predictors of short - term functional decline in survivors of nursing home - acquired lower respiratory tract infection. *Journal of Gerontology Series A Biological Sciences and Medical Sciences,* **58,**(1), 60-67.  Bos, J., Smithee L. & MCClare B. (2005). Fatal necrotizing colitis following a food bourne outbreak of enterotoxigenic clustridium perfringens type A infection. *Clinical Infectious Diseases,* **40,**(10), e78-e83.  Bosshard W., Dreher R., Schnegg J. & La, C.J. (2004) The treatment of chronic constipation in elderly people: An update. *Drugs & Aging,* **21,**(14), 911-930.  Breuer B. & Anderson, R. (2000) The relationship of tamoxifen with depression, dementia and dependence in ADL in elderly nursing home residents. *Women Health,* **31,**(1), 71-85.  Briggs, S., McGuiness C., Foster M. & Roberts S. (2002) A reservoir for methicillin - resistant Staphylococcus aureus in the Auckland community? *The New Zealand Medical Journal,* **115,**(1162), VI82.  Brummel-Smith K., London M.R., Drew N., Krulewitch H., Singer C.& Hanson L. (2002) Outcomes of pain in frail older adults with dementia. *Journal of the American Geriatrics Society,* **50,**(11), 1847-1851.  Burgio L.D. & Burgio K.L. (1990) Institutional staff training and management: a review of the literature and a model for geriatric long term care facilities. *The International Journal of Aging and Human Development,* **30,**(4), 287-302.  Chen J., March L.M., Schwarz J., Zochling J., Makaoff J., Sitoh Y.Y., Lau T.C., Lord, S.R., Cameron I.D., Cumming R.G. & Sambrook P.N. (2005). A multivariate regression model predicted falls in residents living in intermediate hostel care. *Journal of Clinical Epidemiology,* **58,** (5), 503-508.  Cho C., Alessi C.A., Cho M., Aronow H.U., Stuck A.E., Rubenstein L.Z. & Beck J.C. (1998) The association between chronic illness and functional change among participants in a comprehensive geriatric assessment program. *Journal of the American Geriatrics Society,* **46,**(6), 677-682.  Corcoran M.A. & Gitlin L.N. (2001) Family care givers acceptance and use of environmental strategies provided in occupational therapy intervention. *Physical and Occupational Therapy in Geriatrics,* **19,**(1), 1-20.  Corr S. & Bayer A. (1995) Occupational therapy for stroke patients after hospital discharge: a randomized controlled trial. *Clinical Rehabilitation,* **4,**(291), 296.  Doody R.S., Stevens J.C. & Beck, C. (2001) Review: pharmacological and non - pharmacological interventions improve outcomes in patients with dementia and care givers. *Neurology,* **8,** (56), 1154-1166.  Engberg S. (1997) Treatment of urinary incontinence in home bound older adults: interface between research and practice. *Ostomy Wound Management,* **43,** (10), 18-26.  Engel B.T. (1990) Behavioural treatment of incontinence in the long term care setting. *Journal of the American Geriatrics Society,* **38,**(3), 361-363.  Ersser S., Wiles A., Taylor H., Wade S., Walsh R., Bentley T. (1999). The sleep of older people in hospital and nursing homes. *Journal of Clinical Nursing,* **8,** (4), 360-368.  Fryklund B., Haegggman S. & Burman L.G. (1997) Transmission of urinary bacterial strains between patients with indwelling catheters -- nursing in the same room and in separate rooms compared. *Journal of Hospital Infection,* **36,** (2), 147-153.  Giebbach C. (2005) Characteristics of hospitalised geriatrics patients - a comparison of two cohorts using the AGAST. Bayerischen Julius - Maximillans Universitat zu Wurzburg.  Girman C.J., Chandler J.M. & Zimmerman S.L. (2002) Prediction of fracture in nursing home residents. *Journal of the American Geriatrics Society,* **50,** (8), 1341-1347.  Gitlin L.N., Winter L., Dennis M.P., Corcoran M., Schinfeld S. & Hauck W.W. (2006). A randomized trial of a multicomponent home intervention to reduce functional difficulties in older adults. *Journal of the American Geriatrics Society,* **54,**(5), 809-816.  Haeslar E.J., (2004) Effectiveness of strategies to manage sleep in residents of aged care facilities. *Joanna Briggs Institute Reports,* **2,**(4), 115-183.  Hagsten B., Svensson O. & Gardulf A. (2004) Early individualized postoperative occupational therapy training in 100 patients improves ADL after hip fracture: a randomized trial. *Acta Orthopaedica Scandinavica,* **75,** (2), 177-183.  Halves C., Mor, V., Phillips C.D., Flies, B.E., Morris J.N., Stella – Friedlob E., Greene A.M. & Nenshiel M. (1997) The OBRA – 87 nursing home regulations and implementation of the resident Assessment Installment: effects on process quality. *Journal of the American Geriatrics Society,* **45,**(8), 977-985.  Hanson, D., Macejkovic, C., Langemo D., Anderson, J., Thompson, P. & Hunter S., (2006) Research forum. Perineal dermatitis: a consequence of incontinence. *Advances in Skin & Wound Care,* **19,**(5), 246.  Hunter S., Anderson J., Hanson D., Thompson P., Langemo D. & Klug M.G. (2003) Clinical trial of a prevention and treatment protocol for skin breakdown in two nursing homes. *Journal of Wound, Ostomy & Continence Nursing,* **30,**(5), 250-258.  Hurwitz A. (1989) The benefit of a home exercise regimen for ambulatory Parkinson's disease patients. *Journal of Neuroscience Nursing,* **21,**(3), 180-184.  Igou, J. (1986) Incontinence in nursing homes: research and treatment issues from the nursing perspective. *Clinics in Geriatric Medicine,* **2,**(4), 873-885.  Kalra L., Evans A., Perez I., Knapp M., Swift C. & Donaldson N. (2005) A randomised controlled comparison of alternative strategies in stroke care. *Health Technology Assessment,* **9**(18), pp. iii.  Lewis - Abney K. & Rosenkranz C.F. (1994) Content validation of imposed skin integrity and urinary incontinence in the home health setting. *Nursing Diagnosis,* **5,**(1), 36-42.  Lord S.R., March L.M., Cameron I.D., Cumming R.G., Schwarz K., Zochling J., Sheng J., Makaroff J., Sitoh, Y.Y., Lau T.C., Brnabic A. & Sambrook P.N. (2003) Differing risk factors for falls in nursing home and intermediate-care residents who can and cannot stand unaided. *Journal of the American Geriatrics Society,* **51,**(11), 1645-1650.  Macarthur R.D., Lehman M.H., Currie- McCumber C.A. & Shlaes D.M. (1988) The epidemiology of gentamicin - resistant *pseudomonas aeruginosa* on an intermediate care unit. *American Journal of Epidemiology,* **128,**(4), 821-827.  McDowell B.J., Engberg S., Sereika, S., Donovan N., Jubeck M.E., Weber E. & Engberg R. (1999) Effectiveness of behavioral therapy to treat incontinence in homebound older adults. *Journal of the American Geriatrics Society,* **47,**(3), 309-318.  Messecar D. (2003) Commentary; An exercise and incontinence intervention did not reduce the incidence or cost of acute conditions in nursing home residents. *Evidence Based Nursing,* **6**, 117.  Mohide E.A., Tugwell P.X., Caulfield P.A., Chambers L.W., Dunnett C.W., Baptiste S., Bayne, J.R., Patterson C., Rudnick K.V. & Pill, M. (1988) A randomized trial of quality assurance in nursing homes. *Medical Care,* **26,**(6), 554-565.  Moseley C.B. (1996) The impact of federal regulations on urethral catheterisation in Virginia nursing homes. *American Journal of Medical Quality,* **11,**(4), 222-226.  Nelson D.L. & Glass L.M. (1999) Occupational Therapists' involvement with the minimum data set in skilled nursing and intermediate care facilities. *American Journal of Occupational Therapy,* **53**(4),. 348-352.  Nygaard H.A. (1998) Falls and psychotropic drug consumption in long - term care residents: is there an obvious association? *Gerontology,* **44,**(1), 46-50.  Ouslander J.G. (1986) Development and testing of an incontinence nursing record. *Journal of the American Geriatrics Society,* **34**, 83-90.  Palmer M.H. McCormick K.A., Langford A., Langlois J. & Alvaran M. (1992) Continence outcomes: documentation on medical records in the nursing home environment. *Journal of Nursing Care Quality,* **6,**(3), 36-43.  Perry S., Shaw C., McGrother C., Matthews R.J., Assassa R.P., Dallosso H., Williams K., Brittain K., Azdn U., Clarke M., Jagger C., Mayne C. & Castleden C.N. (2002) Prevalence of faecal incontinence in adults aged forty years or more living in the community. *GUT - Journal of the British Society of Gastroenterology,* **50,**(4), 480-484.  Phillips C.D., Morris J.N., Hawes C., Fries B.E., Mor, V., Nennstiel M. & Iannacchione V. (1997) Association of the Residential Assessment Instrument (RAI) with changes in function, cognition and psychological status. *Journal of the American Geriatrics Society,* **45,** (8), 986-993.  Proctor R., Burns S., Powell H.S., Tarrier N., Faragher, B., Richardson G., Davies L. & South, B. (1999) Behavioural management in nursing and residential homes: a randomised controlled trial. *Lancet,* **354,** (9172), 26-29.  Raz, R., Gronich D., Ben-Israel Y. & Nicolle L.E. (2001) Asymptomatic bacteriuria in institutionalized elders in Israel. *Journal of the American Medical Directors Association,* **2,**(6), 275-278.  Reuben D.B., Wolde-Tsadik G., Pardamen B., Hammond B., Brook G.M., Rubenstein L.Z. & Beck J.C. (1992) The use of targeting criteria in hospitalized HMO patients: results from the demonstration phase of the Hospitalized Older Persons Evaluation (HOPE) study. *Journal of the American Geriatrics Society,* **40,**(5),482-488.  Richards K.C., Beck C., Shue V.M. & Sullivan P.S. (2005) Demographic and sleep characteristics in cognitively impaired nursing home residents with and without severe sleep/wake pattern inefficiency. *Issues in Mental Health Nursing,* **26,**(7), 751-769.  Richardson J.P. & Hricz L. (1995) Risk factors for the development of bacteremia in nursing home residents. **9,**(785), 789.  Saltmarche A., Pringle D.M. & Reid, D.W. (1991) Habit retraining: an incontinence study that leaked. *Neurourology and Urodynamics,* **10,** (4), 413-414.  Savik K., Fan Q., Bliss D. & Harms S. (2005) Preparing a large data set for analysis: using the Minimum Data Set to study perineal dermatitis. *Journal of Advanced Nursing,* **52,**(4),399-409.  Schlenker R.E., Powell M.C. & Goodrich G.K. (2005) Initial home health outcomes under prospective payment. *Health Services Research,* **40,**(1), 177-193.  Scuvee - Moreau J., Kurz X., Dresse A. & Nades Group. (2002) The economic impact of dementia in Belgium: results of the National Dementia Economic Study Group (NADES). *Acta Neurologica Belgica,* **102,**(3), 104-113.  Silverblatt F.J., Tilbert C., Mikolich D., Blazek-D'Arezzo J., Alves J., Tack M. & Agatiello P. (2000) Preventing the spread of vancomycin-resistant enterococci in a long-term care facility. *Journal of the American Geriatrics Society,* **48,**(10), 1211-1215.  Simmons S.F., Alessi, C. & Schnelle J.F. (2001) An intervention to increase fluid intake in nursing home residents: prompting and preference compliance. *Journal of the American Geriatrics Society,* **49,**(7), 926-933.  Sims R.V., Hauser R.J., Adewale A.O., Maislin G., Skeie S., Lavizzo – Mourey R.J. & Rubin H. (1995) Acute gastroenteritis in three community based nursing homes. *Journal of Gerontology Series A Biological Sciences and Medical Sciences,* **50,** (5), M252-M256.  Smith D.M. (1995) Pressure ulcers in the nursing home. *Annals of Internal Medicine,* **123,**(6), 433-442.  Steel J. (1995) Minimising the cost of urniary incontinence in nursing homes. *Pharmacoeconomics,* **7,**(3), 191-197.  Steiner J.F. & Kramer A.M. (1997) Development and validation of a clinical prediction rule for prelonged nursing home residence after hip fracture. *Journal of the American Geriatrics Society,* **45,**(12), 1510-1514.  Stoddart H., Whitley E., Harvey I. & Sharp D. (2002) What determines the use of home care services by elderly people? *Health & Social Care in the Community,* **10,**(5), 348-360.  Stoecklin M.T., Weaver K., Haan, M.N., Gilmer D.F., Beck J. & Francis D. (1998) Residential care settings in California: what health care professionals need to know. *Annals of Long Term Care,* **6,**(11), 342-346.  Subak L.L., Quesenberry C.JR., Posner S.F., Cattolica E. & Soghikian K., 2002. The effect of behavioral therapy on urinary incontinence: a randomized controlled trial. *Obstetrics & Gynecology,* **100,** (1), 72-78.  Tannenbaum C. & Dubeau C.E. (2004) Urinary incontinence in the nursing home: practical approach to evaluation and management. *Clinics in Geriatric Medicine,* **20,**(3), 437-452.  Tariot P.N. (2003) Medical Management of Advanced Dementia. *Journal of the American Geriatrics Society,* **51**, 305-313.  Thom D.H., Haan M.N. & Van Den Eeden S.K. (1997) Medically recognized urinary incontinence and risks of hospitalization, nursing home admission and mortality. *Age & Ageing,* **26,**(5), 367-374.  Tsuji, I., Whalen S. & Finucane T.E. (1995) Predictors of nursing home placement in community-based long-term care. *Journal of the American Geriatrics Society,* **43,**(7), 761-766.  Viskum B. (1992) Accidental falls in nursing homes. A study of the extent and circumstances of accidental falls in nursing homes. *Ugeskar Laeger,* **154,**(43), 2950-2955.  Von Koch L., Holmqvist L.W., Kostulas V., Almazan J. & Pedro-Cuesta J.D. (2000) A Randomized Controlled Trial of Rehabilitation at Home After Stroke in Southwest Stockholm: Outcome at Six Months. *Scandinavian Journal of Rehabilitation Medicine,* **32,**(2), 80-86.  Wang P.S., Levin, R., Zhao S.Z. & Avorn J. (2002) Urinary Antispasmodic Use and the Risks of Ventricular Arrhythmia and Sudden Death in Older Patients. *Journal of the American Geriatrics Society,* **50,**(1), 117-124.  Warnke A., Meyer G., Bender R. & Muhlhauser I. (2004) Predictors of adherence to the use of hip protectors in nursing home residents. *Journal of the American Geriatrics Society,* **52,**(3), 340-345.  Warrilow M., Williams D. & Guest, J. (2004) The introduction of a 'trial without catheter' model in primary care. *British Journal of Nursing (BJN),* **13,**(17), 1035-1040.  Wu, J. & Baguley I.J. (2005) Urinary retention in a general rehabilitation unit: prevalence, clinical outcome, and the role of screening. *Archives of Physical Medicine & Rehabilitation,* **86,**(9), 1772-1777.  Yip Y.B. & Cumming R.G. (1994) The association between medication and falls in Australian nursing home residents. *The Medical Journal of Australia,* **160,**(1),14-18.  Young J.B., Robinson M., Chell S., Sanderson D., Chaplin S., Burns S, E. & Fear J. (2005) A whole system study of intermediate care services for older people. *Age & Ageing,* **34,**(6),577-583.  Yu, L.C., Johnson K., Kaltreider D.L., Hu, T., Brannon D. & Ory, M. (1991) Urinary incontinence: nursing home staff reaction toward residents. *Journal of Gerontological nursing,* **17,**(11),34-41. |
| --- |
